# Empirical evaluation of computational models of lightness perception

**DOI:** 10.1038/s41598-022-22395-7

**Published:** 2022-12-21

**Authors:** Predrag Nedimović, Sunčica Zdravković, Dražen Domijan

**Affiliations:** 1 Laboratory for Experimental Psychology, Department of Psychology, Faculty of Philosophy, University of Belgrade, Belgrade, Serbia; 2 Laboratory for Experimental Psychology, Department of Psychology, Faculty of Philosophy, University of Novi Sad, Novi Sad, Serbia; 3 Department of Psychology, Faculty of Humanities and Social Sciences, University of Rijeka, Rijeka, Croatia

**Keywords:** Psychology, Computational models

## Abstract

Lightness of a surface depends not only on its physical characteristics, but also on the properties of the surrounding context. As a result, varying the context can significantly alter surface lightness, an effect exploited in many lightness illusions. Computational models can produce outcomes similar to human illusory percepts, allowing for demonstrable assessment of the applied mechanisms and principles. We tested 8 computational models on 13 typical displays used in lightness research (11 Illusions and 2 Mondrians), and compared them with results from human participants (N = 85). Results show that HighPass and MIR models predict empirical results for simultaneous lightness contrast (SLC) and its close variations. ODOG and its newer variants (ODOG-2 and L-ODOG) in addition to SLC displays were able to predict effect of White’s illusion. RETINEX was able to predict effects of both SLC displays and Dungeon illusion. Dynamic decorrelation model was able to predict obtained effects for all tested stimuli except two SLC variations. Finally, FL-ODOG model was best at simulating human data, as it was able to predict empirical results for all displays, bar the Reversed contrast illusion. Finally, most models underperform on the Mondrian displays that represent most natural stimuli for the human visual system.

## Introduction

**Figure 1:**
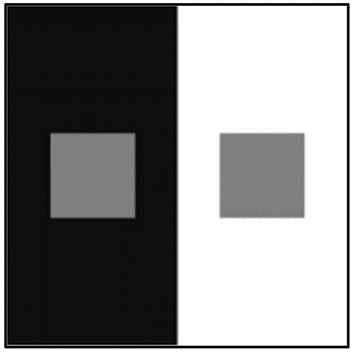
Influence of context on target lightness: two gray targets, despite having the same luminance, appear different in lightness.

Visual system is designed to extract a large amount of information from light registered at retina. In a fraction of a second, it builds a fairly accurate representation of external environment that enable organism to successfully navigate through the world. Despite this success, there are instances where our visual representation departs from the physical distribution of emitted light. Figure 1 depicts such an example in which the two squares of equal luminance appear unequally gray because they are surrounded by different backgrounds.

In the demonstration on Fig. 1 the visual characteristic under scrutiny is color, something visual system is uniquely specialized for. Furthermore, in this example of an illusion we do not process other dimensions of colors (such as hue, since the display is black and white) but only lightness. Lightness illusions, like many others (in vision and other senses), offer important clues about the design of biological machinery that supports perception.

There are only a handful of scientific theories of human vision that deal with lightness percepts produced in regular conditions^1,2^ and for them, illusions present specific challenge. These theories are so called ‘mid-level’ theories (for the low/high level theories see Ref.^3^), dealing with a single characteristic of the visual percept, lightness^1,2^. They are very successful in proposing general principles that map properties of the stimuli to the properties of the conscious precept. Above all, they attempt to explain visual system ability to achieve lightness constancy (i.e., veridical perception) in very complex viewing conditions (multiple surfaces and illumination levels), while deviating from constancy with simple displays, most notably illusions (very impoverished stimuli with only a few surfaces of different reflectance).

Lightness theories, therefore originally developed to account for perception of typical visual scenes and objects encountered in environment, propose wide variety of biological and perceptual mechanisms (i.e., lateral inhibition, perceptual grouping, decomposition etc.). However, these models do not offer precise quantitative predictions in each case, and when it comes to illusions, such as those depicted in Fig. 1, they can generally predict size and direction of illusory effect, but again do not provide precise values or computational mechanisms.

In contrast, a substantial number of computational models of lightness perception offers precise quantitative predictions for perceived lightness or brightness of various illusory stimuli (see the description of models and illusions later in the text). Unlike the theories of human vision, computational models are mostly designed to explain illusions such as those depicted in Fig. 1. They offer algorithms inspired by the same biological and perceptual mechanisms (lateral inhibition, perceptual grouping, etc.) but also precise computational values. Unfortunately, most models can successfully predict outcomes only for a limited number of illusory displays that they were originally designed for. Most importantly, they are rarely tested on natural scenes (High-Pass filtering model being exception).

At the moment, it is not possible to make a firm judgment which of the proposed models provide the best account of available lightness phenomena. Direct comparison is difficult because models are often tested on different displays that vary in sizes and assumed luminance values. Moreover, some of the models have gone through an extensive development over time (such as RETINEX or ODOG) so their different versions might not be comparable. For their evaluation, models are often compared to human data obtained in diverse experimental conditions, again making comparison of the model success difficult to review.

Finally, the comparison of models to human data has one more shortcoming. Any biological system provides its best output when it is tested in the conditions in which it has evolved. In the case of visual system, that would be fairly rich visual scene containing many overlapping surfaces under varying illumination. In contrast, visual illusions are impoverished stimuli containing only a small number of uniform surfaces and usually just three luminance levels. Despite their apparent simplicity, illusions seem to defeat the visual system by forcing it to make errors. Thus, it is natural to ask whether the models, which are designed to explain lightness illusions, will generalize to complex stimuli that are optimized for the biological system?

In this study we will test 8 computational models (see details below) that are proved to be successful in dealing with the most important class of lightness and brightness illusions that represent a distinct challenge to those computational models (and also lightness theories). In addition, we will test two displays more optimized for biological observers (and hence easily handled by the theories made for humans). We will evaluate these 8 computational models against human data obtained on the exact same displays used to test models (which for this reason are not necessarily presented in their regular format).

### Illusions

**Figure 2:**
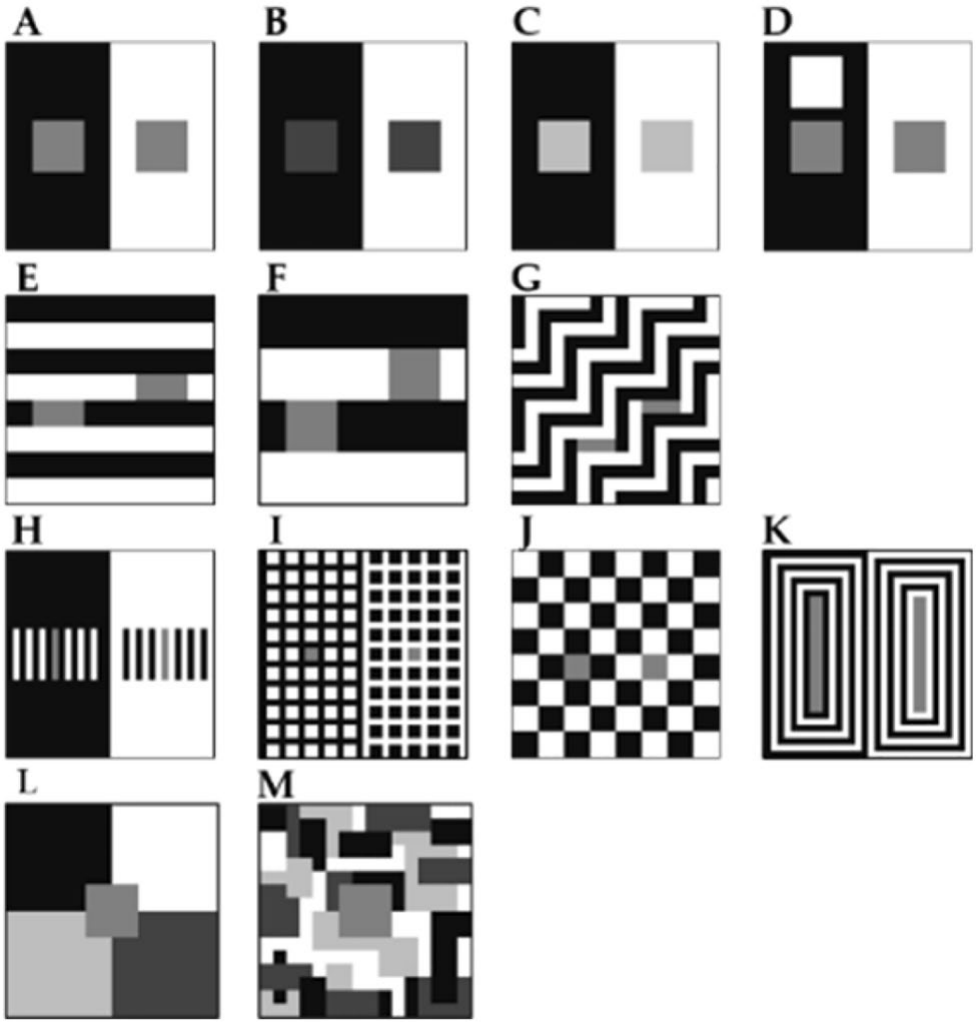
Examples of displays used to test models and human observers: SLC (**A**–**C**), Maniatis variation of SLC (**D**), White’s (**E**,**F**), Wedding cake (**G**), Reversed contrast (**H**), Dungeon (**I**), Checkerboard (**J**) and Bullseye (**K**) illusions, Mondrian displays (**L**,**M**).

Our choice of these particular 11 illusions (Fig. 2) is based on their theoretical and explanatory importance, perceptual processes and significance for the computational models (i.e., some models are directly based on some of these illusions). It is also important to stress that we have modified original (and usual) appearance of these illusory displays to fit the tested models.

*Simultaneous Lightness/Brightness Contrast* (SLC^4^, Fig. 2A) is demonstrating basic illusory effect in lightness: the immediate background can alter the appearance of the target. In this illusion physically equal grey squares appear different against white and black background. Contrast theories^5^ claimed that the white background has a suppressive effect making that target appear lighter, while Anchoring model^2^ proposes that the target on the black background should appear lighter given that it is a highest luminance within its framework. Empirical data back up the latter model. Moreover, Anchoring model easily handles variation in the target reflectance (Fig. 2B,C) correctly predicting larger illusion for the darker targets^6^.

*Maniatis variation of SLC*^7^ (Fig. 2D) however challenges Anchoring Model explanation. The only difference in the display is added white square on the black background. According to Anchoring theory, the grey target is no longer highest luminance within its framework and should appear veridical, i.e., the added white should eradicate the illusory effect. In reality, the effect is drastically weakened but the difference between the two targets is still significant.

*White’s Illusion*^8^ (Fig. 2E) has targets neighboring both white and black background, nevertheless the lightness does not depend on the length of the edge with each region but rather on perceptual assignment of the targets position. Targets forming a group, with what appears to be their background, determines the lightness. The effect was initially explained with the T-junctions^9^.

*Wedding Cake Illusion*^10^ (Fig. 2G) is a clever variation of White illusion that basically produced the same effect, but is made without T-junctions and therefore represents a challenge for that class of explanations.

*Reversed Contrast*^11^ (Fig. 2H) particularly challenges Contrast theories account showing that the target on the white background can actually look lighter than the target on the black, and even lighter than the veridical value, when the target is appropriately grouped with the white flankers.

*Dungeon Illusion* (Bressan and Kramer^12^, Fig. 2I) further challenges Contrast theories showing that grouping can also happen with the background. The net appears to be in front of the grey targets and therefore does not affect their appearance. Just like in the previous case, the target is no longer altered by the neighboring regions but those that constitute its perceptual group.

*Checkerboard Illusion*^13^ (Fig. 2J) was made to demonstrate the effect of spatial resolution of elements size on contrast effect. Depending on the size of the elements the effect flips between contrast and assimilation.

*Bullseye Illusion*^14^ (Fig. 2K) is another example of display that demonstrates assimilation. Just like in the previous case the effect can be flipped, in this case crucial is procedural change (2 AFC vs. matching).

Apart from these 8 illusions we have also included a collage made of gray shades (usually dubbed *Mondrian display*, Fig. 2L,M). The two displays have different number of background surfaces and such increased articulation helps visual system to achieve constancy, a finding easily explained by the lightness theories.

### Computational models

There exists a number of computational models of lightness and brightness perception and it would be a daunting task to evaluate all of them. Thus, we imposed two selection criteria: one conceptual and one practical. On a conceptual level, we omitted models that deal just with a subset of lightness phenomena^15^, or models that gave only qualitative predictions^16^. On a practical side, we restricted our attention to models that came with freely available software code and default parameter set. In this regard, we gratefully acknowledge the help from Alejandro Lerer, Richard F. Murray, and Alan E. Robinson. We refrained from developing our own code of previously published models because there is always a danger of inadvertently committing an error that may distort the model’s output and hamper its evaluation. Thus, we omitted from our evaluation several older filling-in models, for which we were unable to obtain code^17–19^.

Along the same line, we did not include the model of edge integration of Rudd^20–22^ because it incorporates a distance-dependent weighting function which is not specified in sufficient detail. There is a range of options of how such function might be defined and searching through a parameter space to find optimal solution, given our large dataset, seems too demanding. Although this model has been successful in explaining a wide range of lightness data, further work is needed to make its spatial interactions fully automatic. We hope that our work will encourage developers of future models to openly share their source codes in order to make model comparison more transparent and efficient.

Based on the above criteria, we choose to test 8 models of lightness or brightness perception. Next, we provide short overview of each of them.

*High-pass filtering* Shapiro and Lu^23^ suggested that visual system effectively discard unnecessary low-spatial-frequency content in the image when it encodes object appearance, that is, it performs high-pass filtering. On this view, visual system reduces the response to spatial content that is coarser than the size of the attended object. There are many computational procedures that produce high-pass filtered image. One method is to use Fourier transform to represent image in a frequency domain. After removal of low spatial frequencies, inverse Fourier transform is used to reconstruct high-pass filtered image. Another possibility is to directly subtract the blur form the image. In this method, image is first convolved with uniform filter that averages luminance within its window. Next, the output of convolution is subtracted from the original image to obtain high-pass filtered image. Shapiro and Lu^23^ showed that many brightness phenomena including checker-shadow, argyle and dungeon illusions can be explained by this simple process if the size of the convolution filter is set to be equal to the diameter of the test patches.

*RETINEX* is a model inspired by the Wallach’s ratio rule stating that luminance ratio between neighbouring surfaces remains constant under variable illumination. Thus, RETINEX starts from computing ratio between luminance values registered at neighboring pixels. In particular, computation of lightness in RETINEX consists of four steps: ratio, product, reset and average. These steps are iteratively applied across all pixels in the image. Computations are made by following a path, or set of paths, from pixel to neighboring pixel throughout the image. Newer versions of the RETINEX avoid path following and replace it with computationally more efficient spatial comparisons. In a version proposed by McCann^24^, a multiresolution pyramid is created from the input image. Computation of pixel lightness starts at the top level of the pyramid with lowest resolution and the resulting lightness values are propagated to the next lower level in the pyramid. This process continues until lightness values are computed at the pyramid’s bottom level. At all levels in the pyramid, lightness value of each pixel is computed by visiting each of its eight nearest neighbouring pixels in clockwise order and applying four steps described above. It was this McCann^24^ version of RETINEX model that we used in our study.

*Oriented Difference of Gaussian* (*ODOG*) combined multi-scale spatial filtering with response normalization^25,26^. The ODOG model relies on low-level mechanisms that could be implemented in the primary visual cortex. In the first stage, stimulus is convolved with a set of 42 filters. The filters span six orientations and seven scales or spatial frequencies. In the second stage of the model, the global normalization is applied over the output of the first stage summed across scales. The goal of this stage is to equalize the amount of energy at each orientation across the entire visual field. In the original implementation of the ODOG model, unpadded convolution is used. This means that edges of the image are extended by tiling the input pattern. Another possibility, advocated by Robinson et al.^27^, is to pad input image around the edges with gray. This version of the algorithm produces slightly different results and it is labelled as *ODOG-2*.

Robinson et al.^27^ introduced two variants of the ODOG model. They suggested that global normalization used in the original model is not neurally plausible because its computation requires nodes with very large receptive fields. Instead, they proposed locally normalized ODOG (*LODOG*) where orientation energy is normalized within a local window spanning 4° of visual angle. The second variant is a frequency-specific locally normalized ODOG (*FLODOG*). In this variant, normalization is computed separately for each frequency and orientation. The size of the window over which normalization is computed depends on the spatial scale of the filter response. In addition, each frequency channel is normalized primarily by itself. Robinson et al.^27^ showed that FLODOG significantly outperforms ODOG and LODOG in predicting brightness illusions.

*Dynamic decorrelation*: Lerer et al.^28^ proposed a model of brightness perception based on the principle of dynamic decorrelation. They suggested that brightness perception is a consequence of suppressing redundant, that is, predictable information. This is achieved by a dynamic filter that adapts to the spatial structure of luminance patterns in the input image. Lerer et al.^28^ showed that their model is able to correctly predict brightness perception in a large set of illusions and phenomena. It also successfully handles real-world images and images corrupted by noise. The model consists of three processing stages. In the first stage, input image is convolved with a set of Gabor filters with high spatial resolution in the Contrast-only channel and in parallel with Gabor filters with low spatial resolution in the Contrast-luminance channel. Next, the Contrast-only channel serves to compute the local energy map. In the second stage, dynamic filtering is applied on the local energy map in order to decorrelate it, that is, to reduce its redundancy. In the third stage, the model’s output is computed by recovering the image from Contrast-only and Contrast-luminance channels whose responses are gain modulated by the decorrelated energy map.

Murray^29^ developed a probabilistic graphical model, *Markov Illumination and Reflectance* (*MIR*), that decomposes stimulus image into reflectance and illumination component. The model is a conditional random field (a type of Markov random field) that uses belief propagation algorithm to find globally optimal estimates of reflectance and illumination that satisfies a set of statistical assumptions as closely as possible. An example of a statistical assumptions that is built into the model is that reflectance mostly spans the range between 3 and 90%, with a rapid decline in probability outside these limits. Other statistical assumptions are that low illumination is more probable than high illumination and that illumination edges are less common than reflectance edges. By imposing such statistical constraints, Murray^29^ showed that MIR is capable of predicting brightness perception in a number of challenging illusions such as argyle and snake illusion.

There is some debate in the literature regarding the concepts of lightness and brightness. We follow Gilchrist^30^ and define lightness as a perceived surface reflectance whereas brightness refers to a perceived surface luminance. The output of the models of lightness perception such as RETINEX or MIR is given in proportions of reflected light which is mapped onto a range of greys. By contrast, the models of brightness perception such as dynamics decorrelation, ODOG and its variants produce output in unspecified units that correlates with perceived brightness. However, both type of models aim to explain and simulate the same range of phenomena. Thus, it is important to consider them together. Gilchrist^30^ noted that tacit assumption in brightness models is that brightness serves as an input stage for lightness computation. We adopted this view and introduced additional processing stage that operates over the raw output of brightness models. This stage implements “scale normalization” that rescales activity values to the 0–1 range. This procedure transforms brightness estimates into a scale of lightness values similar to the highest-luminance-as-white rule^2^. In addition, we applied the same normalization function to the raw output of the RETINEX and MIR because their outputs are given in different ranges of lightness values (0.20–0.99 for RETINEX, and 0.03–0.90 for MIR). In this way, we were able to directly, and on a common scale, compare output of the lightness and brightness models to human data.

## Methods

### Model simulations

All 8 computational models were run using MATLAB implementations (obtained from Richard Murray for High-Pass, RETINEX, ODOG and MIR; from Alan Robinson for ODOG-2, LODOG and FLODOG; from Alejandro Lerer for DynamicDecorrelation).

All of the 13 stimuli (Fig. 2) were created in MATLAB, on a 16 × 16 matrix grid. Eleven illusion stimuli contained the same three monochromatic regions with values for gray targets: 69 cd/m^2^, black backgrounds: 6 cd/m^2^, and white backgrounds: 145 cd/m^2^. The dark and light SLC had different target values, 25 and 105 cd/m^2^ respectively. Mondrian stimuli had 4 or 34 surfaces, ranging 6–145 cd/m^2^, with targets of 69 cd/m^2^.

Each computational model was tested on all thirteen displays. The parameters of each model were set to default (and were the same for all the displays), and the output of each model was normalized on a 0–1 scale.

### Online experiment with participants

#### Participants

85 participants (mean age 19.3 years, 16 males) took part in an online experiment in exchange for course credits. The study was approved by Institutional Review Board of the Department of Psychology, Faculty of Philosophy, University of Belgrade, Serbia (2021–55), and participants were treated according to the Declaration of Helsinki. Each participant signed an informed written consent prior to the experiment.

#### Stimuli

This was an online experiment, and we could not control participants screens or viewing conditions, so we will present illustrative size and luminance values measured on Lenovo V145 display, 15 inches, with maximum brightness level. These are the same values that were fed into the tested models.

The same 13 displays (Fig. 2), described for model-simulations, were also shown to the participants. Eleven illusion stimuli contained the same three monochromatic regions (R = G = B). The 8 illusion displays that had two targets (both RGB = 127, luminance level: 69 cd/m^2^) and one or more black and white surfaces in the background (RGB 0 and 255, luminance values: 6 and 145 cd/m^2^). The size of the illusory displays was 9 × 9 cm and the size of the target were 2.2 × 2.2 cm for the two SLC displays, 2.2 × 1.1 cm for the White’s, 0.5 × 1.5 cm for the Wedding cake, 0.3 × 2.2 cm for the Reversed contrast, 0.5 × 0.5 cm for the Dungeon, 1 × 1 cm for the Checkerboard and 0.5 × 5 cm for the Bullseye illusion. The three more displays were added, the two variations of the SLC displays with dark gray and light gray targets (RGB 64 and 190, luminances 25 and 105 cd/m^2^ respectively), and a variation of White’s displays that consisted of larger elements with only 4 “stripes” (Fig. 2F, and not 8 “stripes” as in standard depicted on Fig. 2E). The size of the Mondrian was 9 × 9 cm and the size of the target was 2.2 × 2.2 cm. Mondrian stimuli had 4 or 34 surfaces, RGB ranging 0–255 (luminance 6–145 cd/m^2^), with targets of RGB = 127 (69 cd/m^2^).

**Figure 3:**
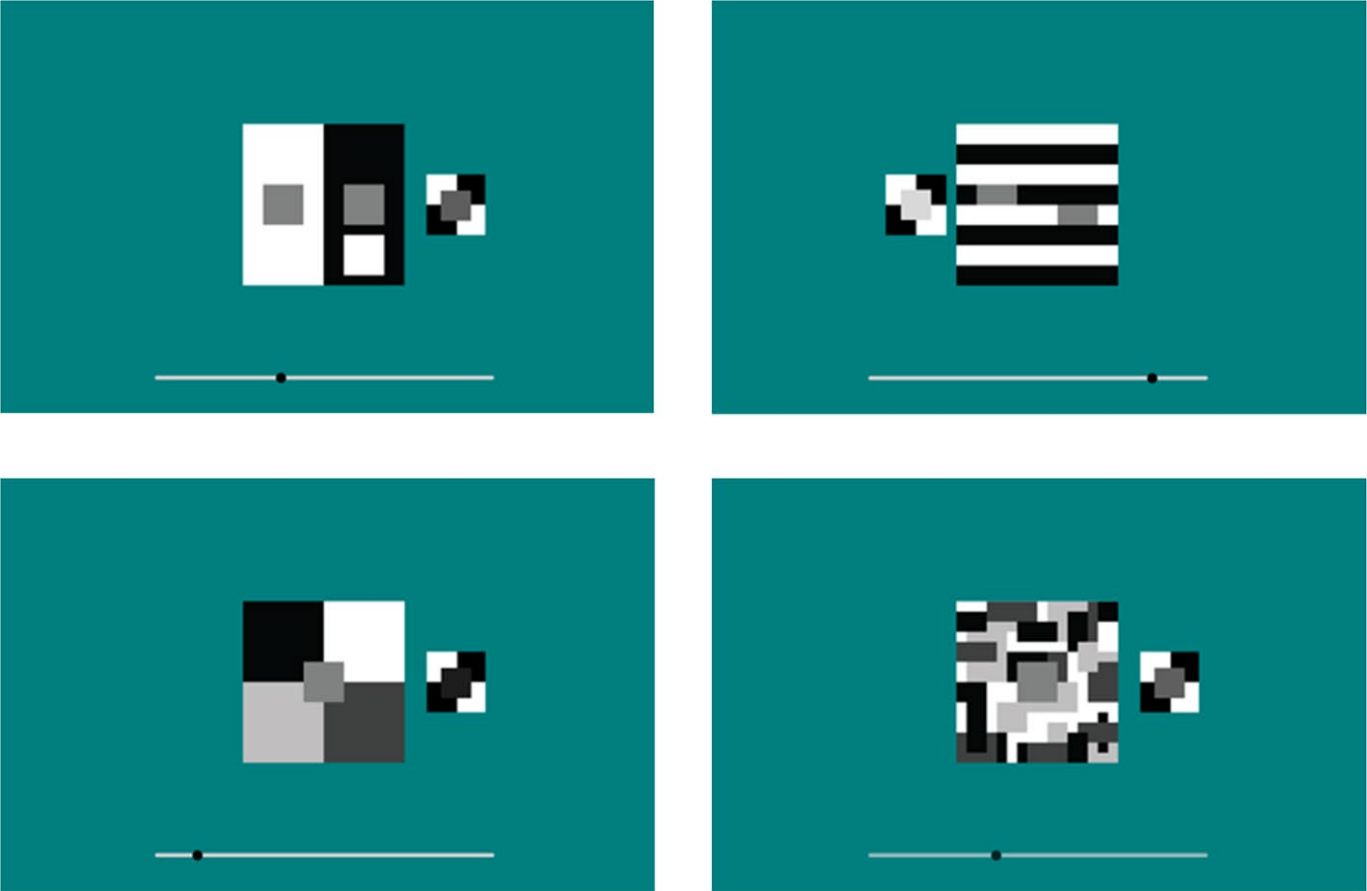
Appearance of 4 screens in our online experiment; different position of the patch signals which target should be matched (left or right, top row), and different position of the bottom slider produces different gray value of the matching patch on the checkerboard background (http://suncicazdravkovic.com/Experiments/Pedja/ClanakDemo/).

Each stimulus was presented in the center of the display (Fig. 3). Next to the stimulus, there was an adjustable square patch on a small black and white checkerboard background. Lightness of the patch was manipulated by the participants via slider on the bottom of the screen (moving a black circle with the mouse). The initial lightness value of the patch was randomized for each stimulus. Patch was presented either to left or right of the stimulus, indicating weather participants should match the color of the left or the right target. The order of stimuli presentation was randomized and each stimulus was presented four times.

Obviously, an online experiment does not offer a laboratory level of screen control. However, we were not focused on absolute but relative values, which depend on RGB values associated with the surface rather than emitted screen luminance. In our conditions, participants compare one target with the matching patch, both presented close to the middle of the screen (ensuring the best luminance values), with the systematically randomize position on the left and right for both (accounting for possible screen anomalies), using mid-gray targets (i.e., staying far away from min and max values that might be questionable on older screens). We were also not interested in small perceptual differences (like JNDs) that could be problematic on uncalibrated screens. Finally, the selected illusions produce robust effects observed in number of different conditions [as reported in various publication, for comprehensive view see Shapiro and Todorović^31^]. Thus, we assume that the differences on participants’ screens would not produce large variations in lightness matches, especially given large sample and large number of repetitions. As it will be seen, our results are comparable to the previous literature and exhibit typical low level of variability in observers’ responses.

## Results

### Human participants: illusion displays

**Figure 4:**
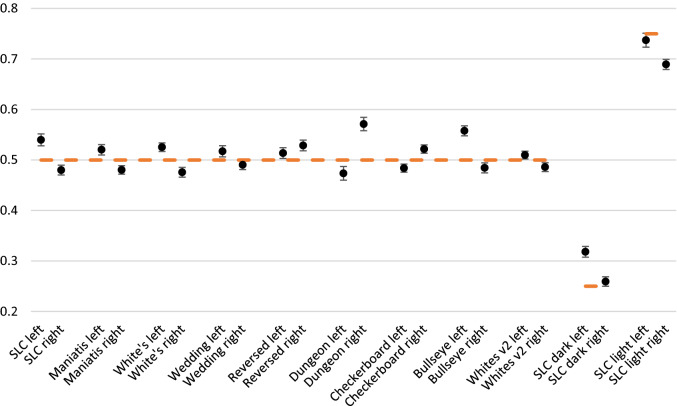
Empirical results from human participants for 11 illusory displays, error bars are 99% confidence intervals.

The results from the human participants for 11 illusory displays are presented in Fig. 4. Obtained results were in RGB values but then normalized on a 0–1 scale to become comparable to the models’ predictions (y axes, Fig. 4). After that transformation, the lightness value for targets is equal to 0.5 on this graph (except for light and dark SLC variations: 0.75 and 0.25 respectively).

Simultaneous lightness contrast display produced lightness illusion in the expected direction: target on the black background (left target) appeared lighter than target on white background (right target), and this difference is statistically significant, as is for all other empirical differences between targets (Table 1). The same is true for three other variations of SLC: dark target SLC, light target SLC and Maniatis variation of SLC. Left target also appeared lighter than the right target for White’s, White’s variation with large stripes and Wedding cake illusions. On the other hand, Reversed contrast, Dungeon, Checkerboard and Bullseye displays produced different effect: target on the left appeared as darker than the target on the right.

**Table 1:** Paired samples t-test results, representing the difference between left and right target matches.

	Md	SDd	SEd	t	df	p	Cohen’s d
SLC	0.060	0.057	0.006	9744	84	< 0.0001	1.057
Maniatis SLC	0.040	0.044	0.005	8285	84	< 0.0001	0.899
White’s	0.050	0.050	0.005	9103	84	< 0.0001	0.987
Wedding cake	0.027	0.054	0.006	4557	84	< 0.0001	0.494
Reversed contrast	− 0.015	0.048	0.005	− 2876	84	0.005	− 0.312
Dungeon	− 0.098	0.071	0.008	− 12,598	84	< 0.0001	− 1.366
Checkerboard	− 0.038	0.043	0.005	− 8103	84	< 0.0001	− 0.879
Bullseye	− 0.073	0.051	0.006	− 13,324	84	< 0.0001	− 1.445
SLC dark	0.059	0.047	0.005	11,606	84	< 0.0001	1.259
SLC light	0.048	0.064	0.007	6930	84	< 0.0001	0.752
White’s large	0.024	0.040	0.004	5464	84	< 0.0001	0.593

These mean values obtained from human participants will be used to evaluate predictions of the 8 computational models.

### Models vs. human participants: illusion displays

#### Simultaneous lightness contrast and Maniatis variation of SLC

**Figure 5:**
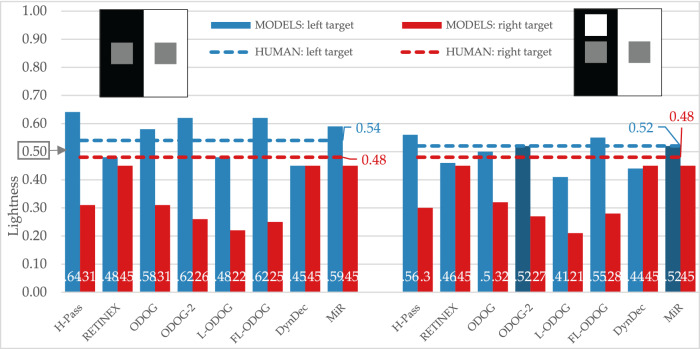
Empirical and model results for SLC and Maniatis variation of SLC displays, no significant difference from human data is annotated with the darker bars. The target lightness value is marked at 0.5 on the Y axis.

Simultaneous lightness contrast display produced lightness illusion in the expected direction: target on the black background (left target) appeared lighter than target on white background (right target) (this difference is statistically significant, as is for all other empirical differences between targets: Table 1). All computational models (except DynamicDecorrelation model) predicted the same direction of effect. It should be noted that all models overestimate the size of predicted effect (FL-ODOG and ODOG-2 being the ones with the largest overestimation (Fig. 5, left).

Maniatis variation of SLC is theoretically important display. Anchoring theory predicts that adding the white surface on the black background should restore veridical perception of that target. However, as Maniatis^7^ observed, just by looking at the display, the illusion persists. Our empirical results confirmed this, as participants perceived left target lighter than the right target, with the illusion size being considerably smaller in comparison to the standard SLC version (Fig. 5, right). Interestingly, the target on the black background is perceived as having the same lightness as in standard SLC, but the target on the black background appears slightly darker. The tested models were also sensitive to Maniatis variation of SLC display predicting smaller lightness difference between the targets. Even more impressive is that computational models correctly predicted target on the black background to be slightly darker for Maniatis variation than in the standard SLC, while there is no significant difference between predictions for target on the white background. Additionally, ODOG-2 and MIR models matched absolute lightness levels for target on the black background (no significant difference from human data is annotated with the darker bars).

#### SLC target luminance

**Figure 6:**
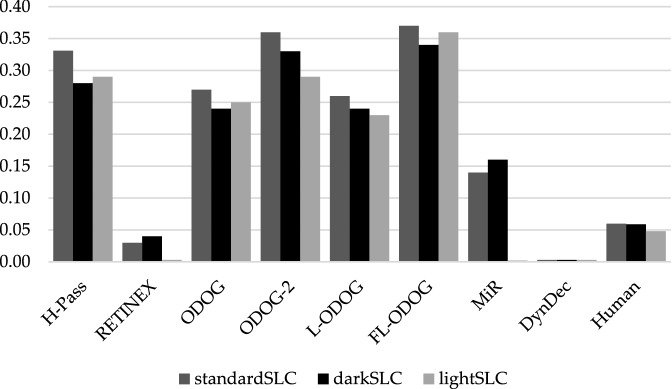
Difference in lightness between left and right target by models and human participants for three target luminance variations of SLC. Values for RETINEX, MIR and DynamicDecorrelation are barely visible because they are at zero.

Given that most models were conceived for SLC and perform well on this illusion, we wanted to perform another stricter test of a very different kind from Maniatis challenge. Economou et al.^11^ showed that the size of SLC illusion increases as target luminance decreases. Unfortunately, this effect was not replicable with the current variation of stimuli. There were no significant differences in any of comparisons (standard vs. dark *t*(84) = − 0.149, *p* = 0.882; standard vs. light *t*(84) = − 1.552, *p* = 0.124; dark vs. light *t*(84) = − 1.526, *p* = 0.131). Although some models (MIR and RETINEX, as seen in Fig. 6) predict typically reported results (e.g., Ref.^6^, when both models and observers are tested on the same stimuli, none of the models predicted the results. RETINEX and MIR do not predict any difference in target lightness for light gray variation of SLC, while DynamicDecorrelation model does not predict any difference in target lightness for any of three SLC variations.

#### White’s and White’s variation

**Figure 7:**
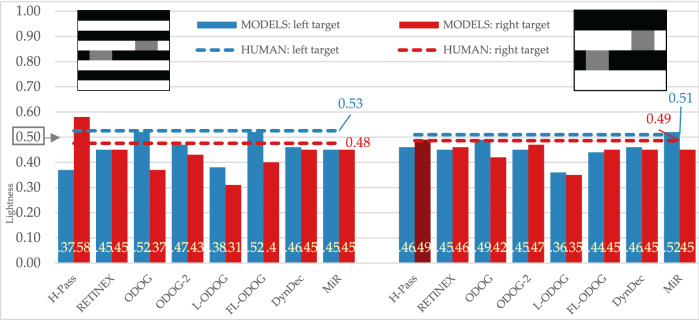
Empirical and model results for standard and large stripes variation of White’s illusions. The target lightness value is marked at 0.5 on the Y axis.

With human participants we obtained expected effect for White’s illusion: target on the left (the one that groups with black background) appears lighter than target on the right (Fig. 7, left). Some computational models were not successful at predicting this effect as they were for SLC displays. RETINEX and MIR models do not predict any effect at all, while HighPass model predicts the reversed effects. However, ODOG models family, along with DynamicDecorrelation models were able to simulate empirically obtained effect.

Empirical results for the White’s variation display (Fig. 7, right) show that left target appeared lighter than the right target, although this effect is smaller than in standard White’s illusion. Computational models produced different results for this variation than for standard display: HighPass, RETINEX, ODOG-2 and FL-ODOG predict reversed effects. This means that only ODOG, L-ODOG, DynamicDecorrelation and MIR models were able to simulate empirically obtained effect.

#### White’s and Wedding cake illusions

**Figure 8:**
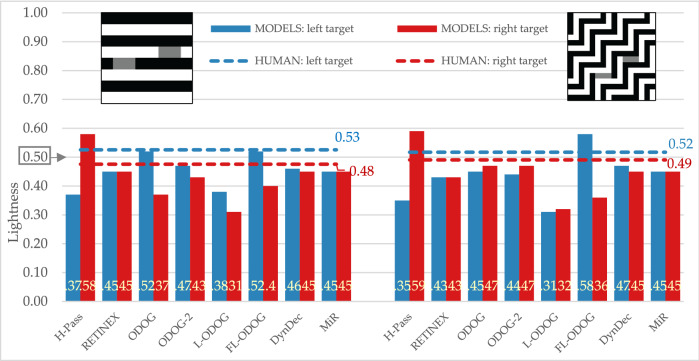
Empirical and model results for White’s and wedding cake illusions. The target lightness value is marked at 0.5 on the Y axis.

Wedding cake illusion produced the same direction of the effect in our participants as White’s illusion (Fig. 8, left), but the effect is smaller (Fig. 8, right). RETINEX and MIR again predict no effect. HighPass, along with ODOG, ODOG-2 and L-ODOG predict reversed effect. Finally, only FL-ODOG and DynamicDecorrelation models were able to match empirical data.

#### Reversed contrast and Dungeon illusions

**Figure 9:**
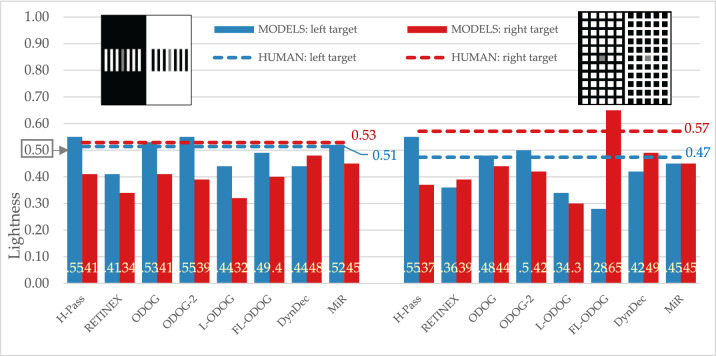
Empirical and model results for Reversed contrast and Dungeon illusions. The target lightness value is marked at 0.5 on the Y axis.

Reversed contrast illusion produced expected effect as our participants perceived left target (on the black background, surrounded by white inducers) as being significantly darker than the right target (Fig. 9, left). Only DynamicDecorrelation model predicted this obtained effect, while all other models predicted standard contrast effects.

Dungeon illusion display produced overall largest effect size out of all tested stimuli (Fig. 9, right). Participants perceived left target (on the black background, surrounded by white net) as darker than the right target. Again, DynamicDecorrelation model predicted this effect, along with FL-ODOG and RETINEX models. Other models either predicted no effect (MIR), or predicted standard contrast effects.

#### Checkerboard and Bullseye illusions

**Figure 10:**
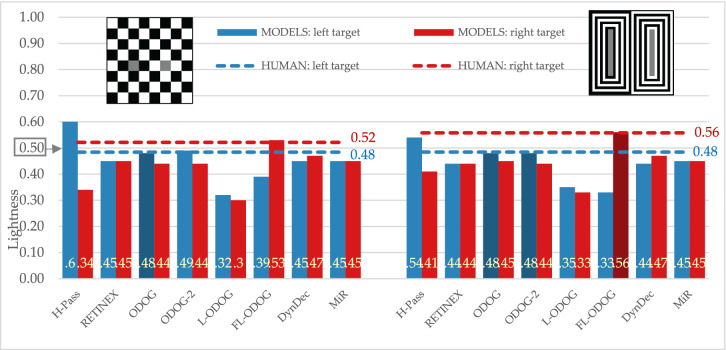
Empirical and model results for Checkerboard and Bullseye illusions. The target lightness value is marked at 0.5 on the Y axis.

When viewing the checkerboard illusion display, participants perceived left target (surrounded by black inducers) as darker than the left target (Fig. 10, left). Only FL-ODOG and DynamicDecorrelation models were able to predict this effect. Other models either predicted no effect (RETINEX and MIR), or predicted standard contrast effects. Finally, Bullseye illusion produced expected lightness effect as left target (surrounded by black inducer) was perceived as darker than the right target by the participants (Fig. 10, right). Model predictions were same as for checkerboard display, since only FL-ODOG and DynamicDecorrelation models were able to predict this effect.

### Models vs. human participants: Mondrian displays

**Figure 11:**
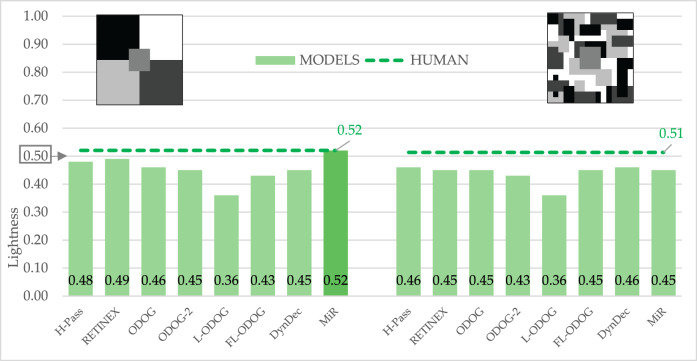
Empirical and model results for two Mondrian displays. The target lightness value is marked at 0.5 on the Y axis.

Finally, we tested two Mondrian stimuli that were shown to produce high level of lightness constancy in human participants. Our results show (Fig. 11) that participants exhibit high levels of lightness constancy for both displays. As expected, the constancy is higher for the Mondrian with higher level of articulation, and that difference is significant (*t*(84) = − 3.156, *p* = 0.002).

One sample t-test showed that only MIR model was able to match absolute lightness levels for first Mondrian display. All other model predictions were significantly different from empirical results. This means that the models were not able to match absolute lightness levels, and consequently were not able to predict these high levels of lightness constancy. Comparing among the models we can see that L-ODOG model predictions deviated the most from the empirical data, while the MIR model was overall best as it matched the absolute lightness levels for our first display. Most importantly, the models seem to considerable fail on the last display, one best handled both by human visual system and theories made on human data.

However, if we look at the results for both Mondrian displays together, most models (apart from L-ODOG, FL-ODOG and DynamicDecorrelation) predicted lower values on the right graph of Fig. 11. This is consistent with the human data. We can say that models are again doing exactly what they are design to do, correctly predicting the difference between the two target values embedded into two different backgrounds. Quantitatively these results are acceptable. Qualitative description is however very different: by slightly lowering reported lightness, human observers are approaching lightness constancy as the observed displays become more complex (i.e., more articulated). It seems that models simply exaggerate the lightness difference between the two targets producing larger errors than what would be expected from empirical observations.

Models’ performance in Mondrians points to an important limitation that will need to be addressed in future computational studies. Human visual system is adapted to process rich displays such as Mondrians and it produces large errors only in simple impoverished displays such as those depicted in Fig. 2A–K. By contrast, models are often tailored to account for brightness perception in these simple displays and, as Fig. 11 clearly demonstrates, they breaks down in more complex and more natural stimulation.

Table 2 provides a summary of all of our results, specifying which models can predict illusions’ directions.

**Table 2:** Summary of findings. Circle indicates that the model matched the direction of illusion.

	SLC	Maniatis	Whites	Dungeon	Wedding	Checker	Bulls	Reversed	Mondrians	
HPass	●	**●**	**×**	**×**	**×**	**×**	**×**	**×**	**×**	2
MIR	●	●	**×**	**×**	**×**	**×**	**×**	**×**	**×**	2
RETINEX	●	●	**×**	●	**×**	**×**	**×**	**×**	**×**	3
ODOG	●	●	●	**×**	**×**	**×**	**×**	**×**	**×**	3
ODOG-2	●	●	●	**×**	**×**	**×**	**×**	**×**	**×**	3
L-ODOG	●	●	●	**×**	**×**	**×**	**×**	**×**	**×**	3
DynDec	**×**	**×**	●	●	●	●	●	●	**×**	6
FL-ODOG	●	●	●	●	●	●		**×**	**×**	7
	7	7	5	3	2	2	2	1	0	

## Discussion and conclusions

We tested 8 computational models on 13 typical displays used in lightness research (11 Illusions and 2 Mondrians) and compared them with the lightness matches from 85 human participants. Computational models generate quantitative lightness predictions comparable to human illusory percepts, allowing us to make a rigorous test of the proposed mechanisms and principles. In this study, we moved forward in model evaluation by performing not just qualitative comparisons of the direction of illusions, but we also made quantitative comparisons about the magnitude of illusions. In particular, we examined how models fare in their ability to account for a broad range of illusions by directly comparing models’ output with human data. It should be noted that we attempted to be as much fair as possible to models by designing new versions of some of well-known illusions in order to make them fit requirements of the models. Importantly, the same stimuli submitted to models to generate their predictions were also shown to the participants.

**Figure 12:**
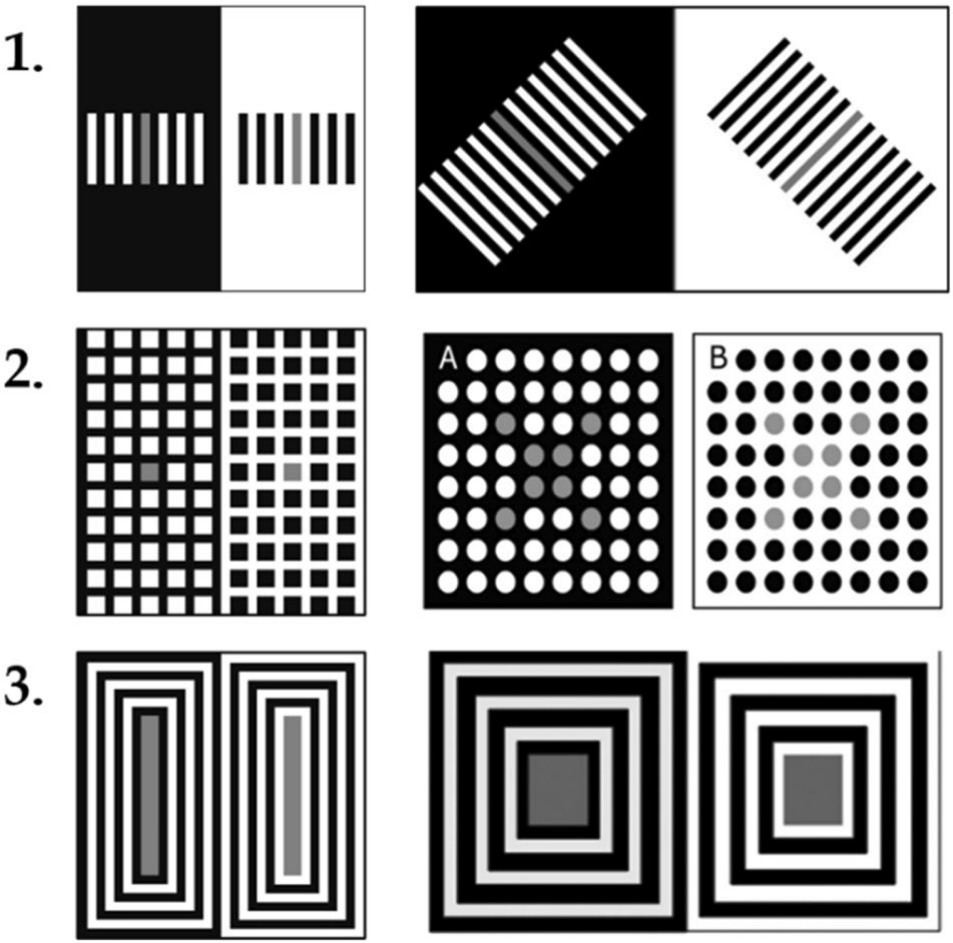
Left column depicts our display in comparison to typical displays used to for testing human observers, presented in the right column used; first row: Reversed contrast, middle row: Dungeon illusion, bottom row: Bullseye illusions.

This optimization might have led to the difference between our human data and previously published data. Figure 12 depicts 3 most evident alterations, where the structure of the display was changed (Reversed contrast, Dungeon illusion, and Bullseye illusions). Other displays were simply reduced in size. The disparities between our data and previous reports were largest for the Reversed contrast, the illusory effect was much smaller than expected. Also, we did not replicate the difference previously found for the SLC with dark targets but this might have also been the consequence of our omnibus procedure whereby a large set of distinct displays were tested within a single session. By contrast, initial result was obtained in isolation from other effects. We also included theoretically intriguing version of SLC developed by Maniatis^7^ and confirmed her observation that illusion persists in this display. To the best of our knowledge, this is the first time that this variation of SLC is experimentally tested.

Majority of models performed well on tested illusions, especially illusions they were particularly designed for. Some models may have achieved even better performance had we changed the model parameters to fit each separate testing, a usual practice when models were tested by their authors. We intentionally avoided this approach, putting models in somewhat unfair position, in order to estimate which model would handle the largest number of visual stimuli, and hence come closest to the general-purpose device such as biological visual system. To this end, we included into our test set two displays that come closest to stimuli typically encountered by biological visual system (Fig. 2L,M). We included some even more demanding tests, like variations of the same illusion, such as SLC with different targets (Fig. 2A–C), or White illusion with different number of stripes (Fig. 2E,F).

All tested models successfully predict some version of regular contrast illusions (e.g., SLC) and this is expected since most models are initially designed with that specific illusion in mind. This means that the models can predict the two important characteristics of the percept, that equal reflectances appear different and the direction of those differences. Models were also sensitive to subtle differences among displays and successfully mimic human data, so most models predict the smaller effect with Maniatis manipulation (Fig. 5), or for the two versions of White’s display (Fig. 7). However, most models do not predict the size of the difference, nor the precise quantitative values. Moreover, models did not perform well on the illusions that revers contrast effects (Fig. 9), DynamicDecorrelation being the only model that can handle direction of the illusion, and FL-ODOG only in the case of Dungeon illusion. Another challenge was assimilation displays, where again only FL-ODOG making successful predictions (Fig. 10). Taken together, FL-ODOG showed best performance in accounting for illusory displays. This suggests that spatial filtering is an important processing stage in generating brightness perception.

Overall, all tested models capture some aspects of human data, but no one can account for all empirical trends. For example, all models (but DynamicDecorrelation) predict SLC, which is not a surprise. Interestingly, all models also correctly predict more subtle effects such as a reduction in the illusion magnitude in a Maniatis version of SLC. However, there are also notable failures related to reverse contrast, assimilation, and Mondrian displays. In that respect, Mondrian displays were particularly interesting because models were not typically designed for this kind of visual stimuli where the absolute value of the perceived surface is crucial. Apart from MIR, with simpler Mondrian, no other models could predict the human data (Fig. 11). It seems that many models, especially those that are based on spatial filtering, rely too much on the process of differentiation of target surface form its background. By contrast, anchoring theory of lightness perception emphasizes the need to integrate local luminance information into a global representation of surfaces that are under common illumination and differentiate it from surfaces that belong to another illumination source^2^. Only MIR incorporates such a global differentiation-integration process but with partial success. Thus, it is still an open question how to best translate principles of anchoring theory into a concrete computational model. Of particular interest would be to integrate perceptual grouping based on common illumination with spatial filtering models such as FL-ODOG as it showed great success in accounting for brightness illusions.

Apart from models tested in our study, literature describes a number of other important models and in Introduction we have justified our choice of these 8 models; it was a mixture of conceptual and practical criteria. Similar was the case with the illusion selection. There is a plethora of known lightness illusions, and we chose (1) the simple ones that could be used for all the models and in the online experiment, (2) those that had practical importance, foremost those for which the models were designed for, and (3) theoretically important for model testing. The last principle means that we submitted models to the three main classes of illusory lightness effects: contrast, reverse contrast, and assimilation.

It would have been useful to test more models on more illusions but this initial study already tested sufficiently large collection of displays and revealed notable weaknesses of various models. Most models correctly predict the direction of illusory effect (that is, which target will appear lighter), but only some can predict the exact strength of illusion (the difference between the two targets). Many models cannot handle the subtler effects such as the differences between contrast and reverse contrast, or contrast and assimilation, and the variations of the same illusion (changes in the target luminance or the spatial frequency). Hence, this study already produced enough information relevant for the future model development.

To conclude, our comprehensive comparisons of computational models with human data suggest that, despite great progress achieved by recent statistical models like MIR or DynamicDecorrelation, we still lack a full understanding of how visual cortex accomplishes such a deceptively simple feat of assigning lightness values to surfaces.

## Supplementary Information

Supplementary Information.

## Data Availability

All data generated or analysed during this study are included in this published article and its supplementary information files. All data are also available here.
